# Lipid and immune abnormalities causing age-dependent neurodegeneration and Parkinson’s disease

**DOI:** 10.1186/s12974-019-1532-2

**Published:** 2019-07-22

**Authors:** Penelope J. Hallett, Simone Engelender, Ole Isacson

**Affiliations:** 1 Neuroregeneration Research Institute, McLean Hospital/Harvard Medical School, Boston, USA; 2 Present Address: Department of Biochemistry, Rappaport Faculty of Medicine and Research Institute, Technion-Israel Institute of Technology, 31096 Haifa, Israel

**Keywords:** Lipids, Immune, Inflammation, Neurons, Microglia, Astroglia, Lysosome, GBA, α-Synuclein, Tau, Apolipoprotein, APOE

## Abstract

This article describes pathogenic concepts and factors, in particular glycolipid abnormalities, that create cell dysfunction and synaptic loss in neurodegenerative diseases. By phenocopying lysosomal storage disorders, such as Gaucher disease and related disorders, age- and dose-dependent changes in glycolipid cell metabolism can lead to Parkinson’s disease and related dementias. Recent results show that perturbation of sphingolipid metabolism can precede or is a part of abnormal protein handling in both genetic and idiopathic Parkinson’s disease and Lewy body dementia. In aging and genetic predisposition with lipid disturbance, α-synuclein’s normal vesicular and synaptic role may be detrimentally shifted toward accommodating and binding such lipids. Specific neuronal glycolipid, protein, and vesicular interactions create potential pathophysiology that is amplified by astroglial and microglial immune mechanisms resulting in neurodegeneration. This perspective provides a new logic for therapeutic interventions that do not focus on protein aggregation, but rather provides a guide to the complex biology and the common sequence of events that lead to age-dependent neurodegenerative disorders.

## Introduction

It is now evident from genetic variant analysis that, lipid and lipid transport, autophagic-lysosomal and inflammatory pathways carry significant and impactful genetic risk for age-dependent neurodegenerative diseases [1–3]. However, it is less clear how their biological interactions lead to the pathology seen in many age-related neurodegenerative disorders, such as Parkinson’s disease (PD), Alzheimer’s disease (AD), and dementias. For example in PD, the first genetic identification of Mendelian genetic human cases involved proteins such as α-synuclein (*SNCA*), *LRRK2*, parkin, *PINK1*, and *DJ-1* [4–13]. Prominently, early discoveries uncovered mutations in the α-synuclein gene in rare families with autosomal dominant PD and the presence of α-synuclein in Lewy bodies in both genetic and sporadic disease [14]. Genome-wide associated studies (GWAS) have also established gene variants associated with lysosomal pathways, lipids, lipoproteins, and inflammation as risk factors for PD, including *GBA1*, *DGKQ*, *LAMP1*, *SCARB2*/LIMP-2, glycoprotein NMB (*GPNMB*), and *HLA* [2, 15]. Critically, it has been shown that the majority of sporadic PD cases (56%) carry at least one putative damaging variant in a lysosomal storage disorder gene, and 21% of patients carry multiple alleles [16]. For these reasons and the pathobiology described in this article, PD clinical trials have begun to try to alleviate the lysosomal dysfunction caused by GBA deficiencies and glycolipid changes in PD patients (ClinicalTrials.gov Identifiers NCT02906020, NCT02941822, NCT02914366).

### Glycolipid changes may be causative of the proteinopathy in Parkinson’s disease

Many neurological diseases such as AD, PD, and the ataxias are conventionally linked to the intracellular accumulation and aggregation of proteins. In the case of PD, α-synuclein and the formation of Lewy bodies has been linked to the disease. However, the key scientific and conceptual question is whether the rare familial hereditary α-synuclein protein abnormalities are representing the same sequence of pathological events that are seen in other genetic causes of PD, or the vast majority of sporadic cases. Significant data shows that lipid vesicles, membrane fragments, and cytoskeletal elements are all found within the α-synuclein-coated Lewy body aggregates at the end-stage of disease in post-mortem PD brain [17], perhaps indicating a generalized cellular dysfunction prior to protein deposition in this disease. Relevant to such cellular dysfunction, brain glycosphingolipid substrate levels are elevated in the brain with increasing age, as well as in sporadic PD [18, 19]. Complex glycolipids are located in the plasma and intracellular membranes of mammalian cells and are particularly enriched in the brain where they have essential roles in cell-cell and cell-matrix interactions, as well as in cell adhesion, modulation of membrane receptors, and signal transduction [20]. As discussed in this article, the physiological burden of increased glycolipid levels in neurons in the aging brain and in PD is therefore likely to influence many cellular organelles and pathways, including lipid membranes, vesicle transport, protein:protein and protein:membrane interactions, autophagic clearance, and neuroinflammation.

### How does varying lipid abnormalities over time lead to different diseases?

**Fig. 1:**
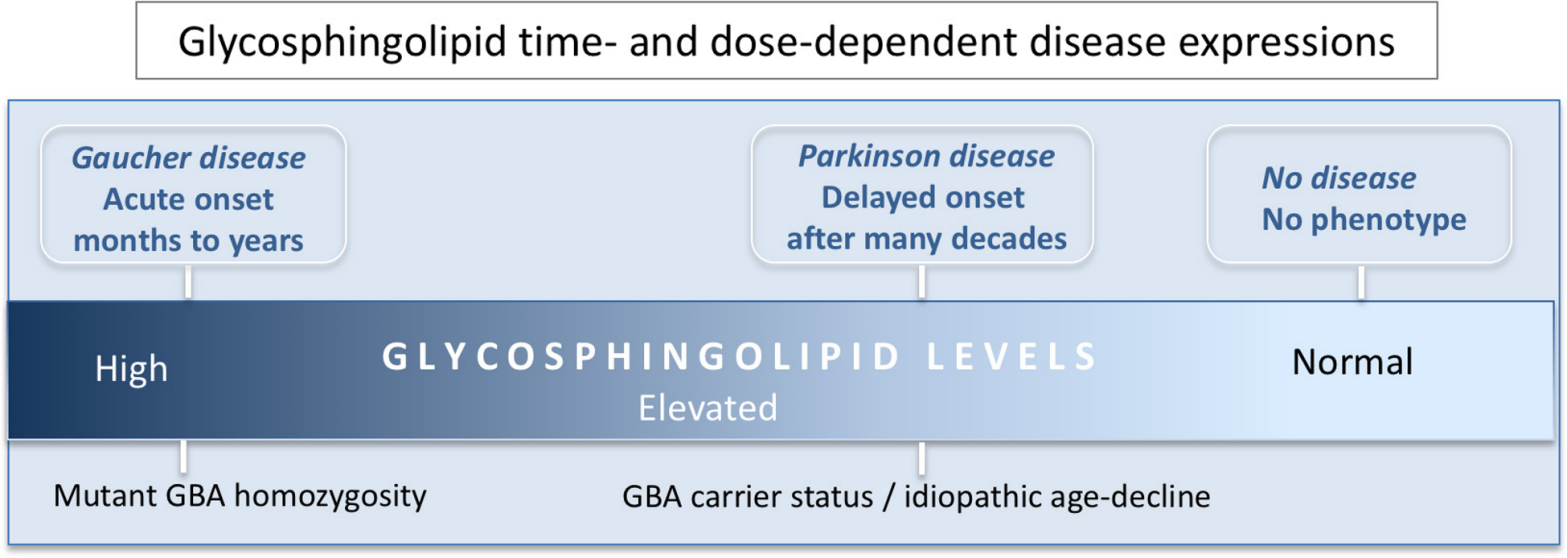
Time and glycosphingolipid dose-dependent phenotypic disease expressions. *GBA1* homozygous or compound heterozygous mutations cause Gaucher disease with onset in early life. Such mutations cause major reductions in glucocerebrosidase (GCase) enzymatic activity and massive accumulation of sphingolipid substrates (glucosylceramide and glucosylsphingosine), typically leading to fatal organ failures particularly of the liver and spleen. In the case of Parkinson’s disease (PD), with or without heterozygous *GBA1* mutations, disease prevalence is in late life (above age 65) and approaches 1–3% of the population. In PD, GCase activity is typically reduced by ~ 50% in the brain and blood with a moderate elevation of glycosphingolipids. Data from post-mortem human brain tissue and in mouse brain also shows that GCase activity is gradually decreased in the brain during normal aging at levels that mirrors genetic GCase haploinsufficiency. Presumably, this contributes to increased risk in all humans with age for PD

Usually presenting clinically by a very early age, homozygote and biallelic *GBA1* mutations in Gaucher disease cause glucocerebrosidase (GCase) loss of function with organ failures of the liver, spleen, and bone [21]. GCase is a lysosomal enzyme involved in the metabolism of glycosphingolipids [22]. The loss of GCase enzyme function resulting from disease-causing *GBA1* mutations occurs through destabilization of protein folding or by preventing synthesis of the full-length protein through frameshifts. Gaucher disease patients without primary neurological manifestations typically have some residual GCase activity and are classified as having non-neuronopathic (type 1) Gaucher disease. Acute neuronopathic Gaucher disease (type 2) affects patients in infancy or even prenatally, with severe neurodegeneration and early lethality [21], and as a result of severe or null mutations in *GBA1*, these patients have little or no GCase activity. Clinically, chronic neuronopathic (type 3) Gaucher disease patients show a degree of neurological involvement but have longer survival (past infancy) [21]. A massive accumulation of GBA-related glycosphingolipids, including glucosylsphingosine and glucosylceramide and related metabolite accumulation are found in the brain in type 2 and type 3 Gaucher disease, with glucosylsphingosine elevations in the brain in chronic neuronopathic Gaucher disease from 22 to 51 times normal, and in acute neuronopathic Gaucher disease from 38 to 694 times normal [23]. Massively increased lipid levels in severe childhood lysosomal storage disease create a different pattern of organ and cellular failure than seen in PD and LBD, where relatively modest but sustained lipid level abnormalities create neurodegeneration later in life. In PD patient carriers of Gaucher disease-linked mutations, haploinsufficiency of *GBA1* produces on average a 30–50% reduction of GCase activity, paralleled with increases in glycolipid substrates [24]. Heterozygous mutations in *GBA1* are associated with an earlier onset of disease and a faster rate of cognitive decline in PD and LBD [25–29]. How do these glycolipid changes of the same biochemical origin cause such different pathologies? For Gaucher’s disease, the pattern of degeneration parallels several sphingolipidoses lysosomal storage disorders in that as a primary function, the liver responds to the massive metabolic lipid disturbances, and both the liver and spleen become seriously compromised. However, with a chronic lifelong, or acquired by age, relatively lower dose of the same lipid species, the liver and spleen manage such glycolipid metabolic load, whereas in vulnerable neural systems, neurodegenerative changes occur. In PD, the midbrain dopaminergic and other brain and neuronal regions will fail to manage such chronic low-level elevations of glycolipids over time (Fig. 1) [31, 32].

### Neurons and cellular systems particularly vulnerable to the Parkinson’s disease pathogenic processes

Whereas organ specificity is relevant, for neurodegenerative diseases, it is more critical to understand individual cellular vulnerability to chronic glycolipid changes that ultimately generate the specific pattern of cell loss in the disease. For example, in PD and LBD, age-dependent neuronal vulnerability and regional synaptic and cellular death follows predictable regional patterns that are defined by complex cell, genetic, and tissue interactions [30, 31, 33]. The initial motor symptoms of PD are largely due to the loss of the dopaminergic midbrain neurons. In the midbrain, the vulnerable A9 and less vulnerable A10 dopaminergic neurons in the substantia nigra have different metabolic and gene expression profiles [30, 31, 34], and we have previously shown that providing A9 neurons with genes that are more highly expressed in A10 neurons provides a cellular resilience against PD-related neurodegeneration in vitro and in vivo [30, 31].

The midbrain dopaminergic neurons, in addition to overall anatomical and normal physiological features, have unique gene and protein expression as well as local tissue milieu and glial interactions that are likely to be involved in their selective vulnerability [31, 33, 35] There is evidence indicating that human dopaminergic neurons in the substantia nigra are subjected to increased oxidative stress during their lifetime as they have increased levels of protein and lipid oxidation products when compared to other regions in the brain [36]. This increased oxidative stress is even more evident in PD. Several markers of oxidative stress are present in the substantia nigra of PD patients, including the reduction of glutathione levels, reduced activity of peroxidase and catalase, and even greater increase of lipid and protein oxidations products [37]. Mitochondrial complex I subunits and activity are decreased in PD brains [38, 39]. Of great significance for understanding age-dependent and common forms of PD, many types of mitochondrial toxicity or metabolic compromise can by itself generate α-synuclein elevations and even aggregated forms of α-synuclein [40].

### α-Synuclein’s normal vesicular role in synaptic transmission can be changed by pathological accommodation to lipids

α-Synuclein is a synaptic protein that is involved in synaptic release of vesicles in many neuronal systems. Along with β- and γ- forms of the protein, α-synuclein specifically can influence the vesicular pore opening times of many neurotransmitter systems in the brain, including the dopaminergic systems [41]. This protein is involved in synaptic plasticity, and was originally described to be involved in learning paradigms of animals [42]. Of interest to the discussion in this article, we believe that α-synuclein has evolved also for other than vesicular membrane lipid binding functions, and potentially, when elevated, has a toxic gain of function at high levels that can prevent axonal transport and potentially other normal functions of the protein [43, 44]. Normal rodent α-synuclein carries a threonine residue at position 53, analogous to the human A53T mutation [45], and would be expected to have altered lipid binding capacity compared to wildtype human α-synuclein, similar to the human mutant A53T α-synuclein. The interpretation being that when mutant A53T, for example, is present in humans, α-synuclein may have inadequate lipid binding transport capacity for human longevity, with clearly increased risk of PD with this mutation [5]. Human neurons derived from induced pluripotent stem cells (iPSCs) show evidence of large lipid particles accumulating in the cell body in vitro when wild-type α-synuclein is overexpressed [44]. As we will describe below, new data put into the context of previous studies illustrate that the lipid binding domain of α-synuclein and its potential role in lipid transport, in concert with other proteins, could become part of a toxic cascade that ultimately results in PD-like pathology.

**Fig. 2:**
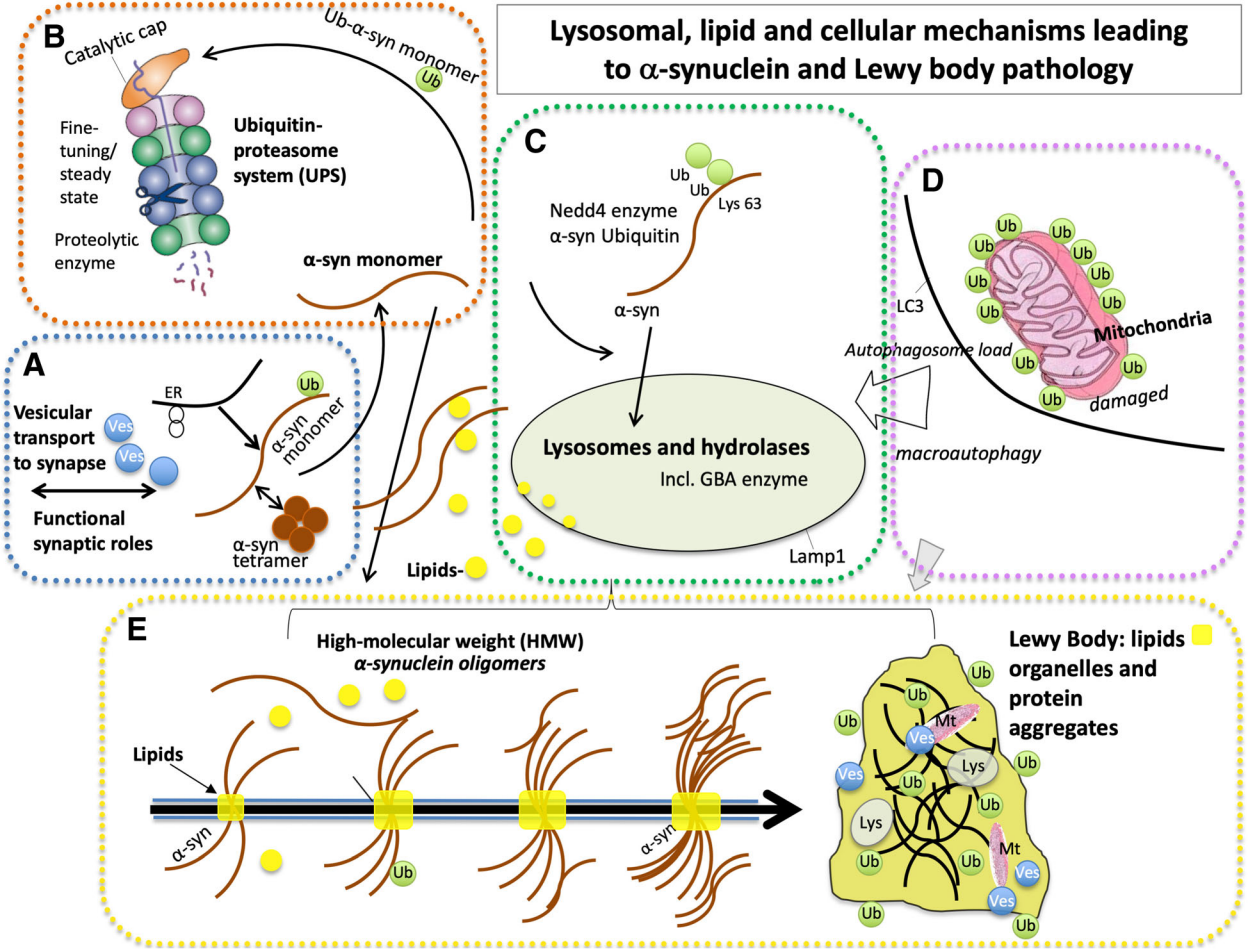
Lysosomal, lipid, and cellular mechanisms leading to α-synuclein and Lewy body pathology. **a** Under normal conditions α-synuclein is a natively structured protein with synaptic and vesicular functions. In addition to monomeric α-synuclein, α-synuclein can exist as physiological α-synuclein multimers (tetramers). α-Synuclein is normally transported to intracellular sites, including the synapse, where it participates in synaptic release functions. **b** Several pathways are involved in the degradation of α-synuclein and maintenance of its steady-state levels, including the ubiquitin-proteasome system (UPS), and **c** endo-lysosomal pathways. Overexpression of the ubiquitin ligase, Nedd4, can rescue α-synuclein toxicity in animal models through increased degradation of α-synuclein through the endo-lysosomal pathway (see text). **c** GBA haploinsufficiency, or deficiencies in other lysosomal enzymes, chaperones or transport proteins, can cause dysfunction of the lysosome in Parkinson’s disease (PD) and Lewy body dementia (LBD). Increased expression or function of lysosomal enzymes including GBA and Lamp1 reduce α-synucleinopathy and can rescue midbrain dopamine neuron degeneration in α-synucleinopathy preclinical models. **d** Damaged mitochondria are degraded through the mitophagy pathway, which can also be disrupted in aging and in familial PD and increases autophagic lysosomal load. **e** As described in this review, the structural similarity of α-synuclein with apolipoproteins suggests an additional role for α-synuclein in lipid transport. An aberrant accumulation of lipids, including glycosphingolipids in aging and in PD, are associated with abnormal lipidation of α-synuclein, and eventually accumulation of α-synuclein into insoluble high molecular weight forms. Mutations in, overexpression of, and loss of function of α-synuclein, contribute to cellular toxicities and pathology, including perturbation of lipid-vesicle trafficking and axonal transport. Recent advanced microscopic evidence shows that the Lewy body is composed of a large lipid core also including cellular organelles including mitochondria, lipids and protein aggregates. ER (endoplasmic reticulum), Lys (lysosome), Mt (mitochondria), Ub (ubiquitin), Ves (vesicle)

In modeling the genetic predisposition to copy number variant (duplication, triplication) and even mutations in the lipid-binding domains of α-synuclein, AAV-mediated overexpression of α-synuclein in the rat substantia nigra highlights the intrinsic differences between vulnerable and less vulnerable midbrain dopamine neurons and generates a relatively selective or sensitive degeneration of the A9 neurons [43, 46, 47]. α-Synuclein can impair microtubule transport systems, and we have found that overexpression of α-synuclein in the substantia nigra using an AAV system leads to an early blockade of anterograde and retrograde motor proteins along the axons, which is paralleled by neuroinflammation in the target region of the AAV-α-synuclein nigrostriatal neurons, and precedes dopamine neuron degeneration [43]. Findings that α-synuclein overexpression produces such microtubule and transport is widely supported in the literature [48–50]. In human neurons derived from iPSCs from PD patients carrying increased dosage of α-synuclein, the resulting α-synuclein oligomerization is associated with mitochondrial axonal transport deficits, reduced ATP levels, and synapse loss [48]. Overexpression of wild-type α-synuclein in human iPSC-derived neurons, is accompanied by massive lipid droplet accumulation [44] and it is perhaps such abnormal α-synuclein-lipid interactions that result in vesicle transport deficits and eventually neuronal degeneration. Significantly, ultrastructural analysis of Lewy pathology in the brain of PD patients shows the presence of lipids, α-synuclein, lysosomes, and mitochondria, and such findings are suggestive of impaired organellar trafficking [17] (Fig. 2a, d).

### Lysosomal dysfunction in PD highlighted by glucocerebrosidase activity reductions

GCase activity is reduced and glycosphingolipid levels are increased, in the brain of PD patients with and without a *GBA1* mutation [18, 51, 52] as well as in the brain in aging [18, 53], and seems to be part of an age-induced phenocopy of the genetic haploinsufficiency of *GBA1* seen in patients [53]. The age-induced changes to lysosomal GCase activity and glycosphingolipid homeostasis would be expected to cause lysosomal dysfunction and accumulation of α-synuclein through several interacting mechanisms. Cross-linking studies have identified a physiological conformation of α-synuclein in α-helically folded tetramers [54], which are hypothesized to be in equilibrium with unfolded α-synuclein monomers [55]. α-Synuclein is degraded by different pathways, including proteasomes, macroautophagy, chaperone-mediated autophagy (CMA), and endolysosomes [56]. Several pathways may participate in maintaining a steady state of physiological α-synuclein as well as involved in its degradation, and their combined or sequential failure may lead to its accumulation in the disease. Supporting this, pharmacological GCase inhibition, knockdown of GBA, or GBA mutations, in mice, primary neuronal cultures and in human neurons, causes the accumulation of glucosylceramide and glucosylsphingosine, decreased lysosomal protein degradation, α-synuclein accumulation and altered multimerization, neuroinflammation, and toxicity [57–65]. Additional mechanisms of glycosphingolipid toxicity could be caused by leaks from lysosomes, disruption of membrane fluidity, direct interactions with α-synuclein, Ca^2+^ dysregulation, and neuroinflammation [57, 66]. A moderate accumulation of glycosphingolipids and altered lipid composition in the plasma membrane and membranous organelles in aging and PD would be expected to be associated with multiple pathophysiological consequences and disrupted neuronal function. A reduction of transport of active GCase to the lysosome may also contribute to decreased lysosomal GCase activity in PD. Lysosomal integral membrane protein type-2 (LIMP-2, also known as SCARB-2) binds GCase to allow for trafficking of GCase from the endoplasmic reticulum to lysosomes [67]. Loss of LIMP-2 in mice leads to reduced GCase activity, lipid storage, α-synuclein accumulation, and gliosis in the brain [68], whereas LIMP-2 overexpression or LIMP-2-derived peptides increase endogenous lysosomal GCase and reduce α-synuclein in α-syn-expressing cell lines [68, 69]. Importantly, overexpression of wild-type GBA in neurons, increasing GCase activity through gene therapy, small-molecule chaperones or activators, and reduction of substrate accumulation, prevents α-synucleinopathy of midbrain dopamine neurons in animal models and human neurons [70–73].

### Protein–lipid interactions in aging and neurological diseases

α-Synuclein can bind lipids through its amphipathic N-terminal amino acid domain, and notably, all α-synuclein mutations that drive familial PD (A30P, E46K, G51D, H50Q, A53T) cluster in the lipid binding domain of α-synuclein. α-Synuclein interacts with several types of lipids, including glycosphingolipids, gangliosides, mono and polyunsaturated fatty acids, and acidic phospholipids [57, 74–81]. We have found that aggregated (proteinase-K resistant) α-synuclein increases with GCase inhibition in vivo [58]. In vitro data using human cells indicate that GCase dysfunction leads to lipid changes that destabilize the postulated tetramer configuration of normal α-synuclein [65]. Moreover, reduction of α-synuclein tetramers in mice leads to excess monomers, accumulation of proteinase-K resistant α-synuclein, intraneuronal accumulation of lipid droplets, and dopaminergic degeneration [55]. Our results from experiments evaluating brain aging in mouse and human brain show reduced GCase activity, elevated glycosphingolipids, and the presence of lipid-associated α-synuclein in the aging brain [18, 19, 53] and may be particularly relevant to understanding the cellular changes that precede α-synuclein linked cell toxicity. Interestingly, aging in mice is associated with aberrant lipidation of α-synuclein and phosphorylated Tau, and their accumulation in dopamine-containing Secretogranin II vesicles [19]. These results [19], together with the ability of α-synuclein to bind lipids and the abundance of lipids in Lewy bodies [17, 82], raise the possibilities that α-synuclein could interact with certain types of lipids under pathological conditions promoting its oligomerization and further aggregation (Fig. 2e), and that lipid perturbations are potentially a primary driver of PD and aging pathology. Consequently, α-synuclein aggregation per se may be a consequence of the disease initiation or typical neuronal toxicity caused by other mechanisms, and so attempts to remove oligomeric aggregated α-synuclein as a treatment of the disease may be futile [83].

### Potentially important mechanisms involving lipid transport and protein pathophysiology

Appropriate lipid transfer between neurons and glia via lipid transporters such as apolipoproteins is essential for maintaining metabolic integrity of neurons. Elevation of reactive oxygen species and mitochondrial dysfunction in neurons leads to elevated lipid production in neurons and subsequent accumulation of lipid droplets, a lipid storage organelle, in glial cells [84]. Impairment of lipid transport from neurons to glia under conditions of oxidative stress results in neuronal damage and leads to neurodegeneration [85, 86].

Apolipoprotein E (apoE) is the most ubiquitous brain lipoprotein and acts as a scaffold for the formation of lipoprotein particles. ApoE transports and delivers cholesterol and other lipids via binding to cell surface apoE receptors. The e4 allele of apoE (*APOE* ε4) is the strongest genetic risk factor for sporadic AD and is found in ~ 60% of AD patients. Compared with apoE3, apoE4 is less able to promote cholesterol efflux and forms smaller CNS lipoproteins [87]. From clinical studies, it is clear that *APOE* ε4 is also linked with increased risk for dementias across the spectrum of Lewy body diseases, including dementia with Lewy bodies, and PD [88–90]. Curiously, mouse apoE is more amyloidogenic than the human apoE isoforms, including apoE4 [91]. Potentially, this could indicate an evolutionary pressure for better lipid handling provided by apoE2 and apoE3 isoforms in humans, since the mouse lifespan is so much shorter than humans.

Previous studies in PD experimental models have addressed and shown an involvement of cholesterol metabolism in α-synucleinopathy, and subsequent amelioration of α-synucleinopathy with cholesterol lowering drugs [92]. Clinical and genetic data support an even broader notion of dysregulation of lipid metabolism in PD and LBD [2, 18]. Relevant to the interactions of α-synuclein and lipids is α-synuclein’s structural and functional similarities to apolipoproteins, and α-synuclein has lipid-related functions [42, 74, 75, 78, 93–98]. The N-terminal region of α-synuclein contains 11-amino acid repeats which mediate membrane interactions of α-synuclein, characteristic of the 11-amino acid repeats that mediate lipid interactions of apolipoproteins. α-Synuclein can induce membrane curvature and tubulation, and form lipoprotein nanoparticles, similar to other apolipoproteins [93, 97]. In in vitro functional assays, α-synuclein can regulate cholesterol efflux in neuronal cells, which was described by these authors as apolipoprotein-like action [96]. The role of α-synuclein functioning like apolipoproteins is also highlighted by the finding that hippocampal apoE4 pathology is exacerbated in mice that carry human apoE4 with a loss of mouse α-synuclein alleles [95]. Moreover, mice deficient in α-synuclein exhibit an elevation in brain neutral lipid levels [99]. Taken together, these studies highlight a premise for α-synuclein’s function as a lipid-carrying molecule.

Variations in triggering receptor expressed on myeloid cells 2 (*TREM2*) are also a significant risk factor for developing late onset AD [100]. TREM2 is a lipid sensor and is expressed by microglia in the brain; TREM2 activation is essential for immune function, and microglia expressing TREM2 may sense lipids on damaged neurons and glia [101]. Indeed, the R47H disease-associated mutation impairs TREM2 detection of lipid ligands [101]. ApoE is also a ligand for TREM2 and can bind to apoptotic neuronal cell surfaces and increase TREM2-mediated phagocytosis by microglia [102–104], suggesting convergence of ApoE lipoprotein and TREM2 on the same pathway. Recently, ApoE has also been shown to reduce inflammation caused by activated C1q by direct protein-protein interaction, and loss of ApoE function produces oxidized lipids that activate the complement cascade [105].

### Multiple lysosomal storage gene dysfunction and disorders create increased risk for Parkinson’s disease

In addition to Gaucher carriers with heterozygote GBA mutations, the increased risk of developing PD is observed in other lysosomal storage diseases with mutations in different enzymes that metabolize lipids in the lysosomes [16, 106, 107] and which are associated with primary accumulation of sphingolipids. α-Synuclein accumulation has also been described in the brain of patients with Sandhoff disease, Fabry’s disease, Krabbe’s disease, and Niemann-Pick disease type C1, as well as in several mouse models carrying mutations in lysosomal storage disorder genes [108–114]. Therefore, the accumulation of several different lipids could contribute to toxicity in neurons. Further studies will be necessary to determine if the lipids of other lysosomal storage disease than Gaucher’s disease also accumulate in PD and in the aging brain. In summary, it appears that accumulation of glycolipids can cause the entire cascade of events associated with PD, including lysosomal blockade, increased intracellular α-synuclein, and fulminant neuroinflammation leading to synaptic degeneration and death (Fig. 2).

Also relevant to PD risk and lysosomal storage disorders, ATP13A2 is a lysosomal ATPase that acidifies the lysosomal lumen and therefore allows the lysosomal proteases to properly function [115]. Mutations in ATP13A2 are associated with Kufor-Rakeb syndrome and young onset PD [116–118], as well as to the lysosomal storage disorder, neuronal ceroid lipofuscinosis. ATP13A2 levels are decreased in dementia with Lewy bodies and PD cases [115, 119]. ATP13A2 depletion has been shown to promote neurodegeneration with endolysosomal abnormalities and reduce the degradation of lysosomal substrates [115, 120–123]. While ATP13A2 knockout from primary neurons and ATP13A2 PD patients-derived fibroblasts lead to lysosomal dysfunction with subsequent accumulation of α-synuclein and toxicity [115, 121], ATP13A2 knockout mice had lysosomal dysfunction and toxicity without the accumulation of α-synuclein [120]. An explanation for these findings could be that there are compensatory mechanisms in living systems that already present a burden of α-synuclein. Interestingly, heterozygous ATP13A2 knockout mice leads to significant gliosis, which over time would likely be detrimental to function of vulnerable neuronal populations [124].

### Dysfunction in mitophagy, cell, and vesicular transport mechanisms in neurodegenerative diseases

Autophagy-dependent removal of damaged mitochondria (mitophagy) (Fig. 2d) is responsible for the selective removal of superfluous and damaged mitochondria under a variety of pathophysiological conditions and is essential to relieve oxidative stress and prevent cytosolic and axonal damage and subsequent cell death. Dysfunction in various steps of the autophagy pathway, for example in autophagosome formation, lysosomal function, autophagic cargo, and autolysosome formation, has been implicated in several neurological and degenerative diseases including PD, AD, polyglutamine diseases, and amyotrophic lateral sclerosis [125]. Abnormalities in mitophagy are described in human sporadic and familial PD patient-derived neurons and fibroblasts (reviewed in [126]). Aging of post-mitotic cells is associated with impaired mitochondrial quality control leading to accumulation of large senescent mitochondria [127]. Of interest, mitochondrial function is compromised in fibroblasts and neurons isolated from PD patients carrying the LRRK2 G2019S mutation [128, 129], and PD patient LRRK2 G2019S mutation fibroblasts show decreased mitophagy by pathways that block the formation of autophagosomes [130].

Relevant for PD and LBD pathology, the targeting of α-synuclein to lysosomes occurs through the fusion with endosomal vesicles (Fig. 2c). Nedd4, an ubiquitin-ligase that has been shown to improve endosomal trafficking of several substrates [131], was shown to ubiquitinate α-synuclein and target it for degradation through the endolysosomal pathway [132]. Consistent with this, the lysosomal inhibitor chloroquine as well as the knockdown of critical endosomal proteins, but not proteasomal inhibitors, prevents the degradation of α-synuclein by Nedd4 [132]. In addition, Nedd4 was found to be increased in dopaminergic neurons containing Lewy bodies [132], suggesting a compensatory increase of this ubiquitin-ligase in conditions of α-synuclein accumulation. In Drosophila and rat models of α-synucleinopathy, Nedd4 overexpression prevents neuronal dysfunction and pathology [133], and NAB2, an activator of the Nedd4 pathway, rescues α-synuclein toxicity in vitro in both yeast and human neuron α-synucleinopathy models [134].

Rab proteins are a family of small GTPase proteins that are important for the vesicular transport from the ER to the Golgi complex but currently they are recognized as having a much broader role in virtually all types of vesicular transport systems (see Fig. 2a), including lysosomes, autophagic and synaptic vesicles [135–137]. Rab proteins were described to be involved in many steps of vesicular transport cycle, including formation of vesicles, vesicular motility along cytoskeleton components, and tethering and fusion to target membranes [138]. Lipids are also transported by Rab proteins, including Rab7, Rab5, Rab1, and Rab18 (reviewed in [138]), and Rabs are dependent on lipids for their function by lipid-induced posttranslational modification (attachment of geranyl group at the carboxyl-terminal cysteine residues) [138, 139]. Dysfunction in membrane trafficking and links to Rab proteins has been described for many neurodegenerative disorders, including AD, PD, Huntington’s disease (HD), amyotrophic lateral sclerosis, and Charcot-Marie-Tooth disease [140]. Our work on Rab function in PD first demonstrated that Rab3B, which is enriched in synaptic vesicles and involved in exocytosis and neurotransmitter release, is intrinsically more highly expressed in A10 dopaminergic neurons, which are less vulnerable to degeneration in PD, than in A9 substantia nigra pars compacta neurons [30, 31]. Gene transfer of Rab3B to A9 dopaminergic neurons in rats increased neurotransmitter content, number and size of synaptic vesicles, levels of presynaptic proteins, and improved vesicular transmitter handling and storage capacity of dopaminergic neurons [30]. In a model of retrograde degeneration of midbrain dopaminergic neurons in rodents induced by oxidative stress (which is potentially associated with lipid disruptions and peroxidation of synaptic vesicles), AAV-mediated overexpression of Rab3B improved motor function and protected dopaminergic neurons from degeneration [30]. Moreover, Rab3B overexpression also improves early metabolic changes caused by α-synucleinopathy in rodents (Hallett and Isacson, unpublished data). Elevated expression of a different Rab protein, Rab1a, normalizes α-synuclein levels in human PD neurons with *SNCA* triplication, potentially through transport to appropriate autophagic systems [141]. Also in the context of PD, Rab modulation of vesicle formation, delivery, tethering, and fusion has been shown to be partially dependent on the kinase activity of LRRK2, a different gene involved in familial PD [142, 143]. Rab proteins could therefore be a pharmacological target for improving vesicular and axonal transport deficits caused by abnormal lipid-protein interactions in PD and LBD.

### Pivotal roles of astroglial and microglial cells in the pathophysiology and inflammatory responses seen in age-related neurodegenerative diseases

Abnormal lipid, protein, and metabolic stressors (for example, due to mitochondrial dysfunction or abnormal lipid metabolism) within PD-susceptible tissue and neurons are likely to signal distress to surrounding cells, including microglia and astrocytes [43, 144]. Neurons are in constant communication with glia. In the context of inflammatory and immunological responses, surprisingly it has been shown that both AD related β-amyloid and α-synuclein can participate in anti-microbial functions [145, 146], and so it is conceivable that the neuroimmune responses are retained and executed in the context of sterile inflammation caused by neuronal dysfunction and damage prior to cell death. Interestingly, the toll-like receptors that are involved in responses to bacteria and viruses have receptor agonists that belong to lipid classes [147] and when severe metabolic and lipid disturbances occur within dysfunctional neurons, such molecules, including fatty acids, may be participating in direct activation of neuroinflammation in brain tissue [148]. A causal link between the accumulation of senescent glial cells with cognitive decline and neuronal loss has been described in a mouse model of tauopathy, and genetic elimination of the senescent glia or pharmacological elimination, prevented cognitive decline and pathology [149].

In yeast and human cells, with extreme calcium, redox, vesicle trafficking, or α-synuclein protein load, all organisms die relatively acutely. However, in the age-dependent degeneration seen in PD (for example), the cells in a living tissue context in many cases are able to homeostatically respond to relatively minor changes in these disruptive mechanisms. In the brain, many different types of microglia (in different proinflammatory or phagocytic states) are in second-to-second surveillance of the overall cellular energy status, and what appears to be a large number of cellular / intracellular signals received. Consequently, immune regulation is an important part of cell growth, degeneration, and death in the more complex organisms that have immune systems. This intricate immune regulation can be altered in positive or negative directions to create abnormal neurodegeneration [150–153]. There is a very significant immunological genetic load that is associated with AD and PD for HLA locus risk and very prominent genetic markers, including the microglial markers that are associated with neurodegeneration.

The role of inflammation in PD has been inferred long ago when patients developed parkinsonism after encephalitis caused by 1918 influenza pandemic, known as von Economo disease [154]. The connection of inflammation and parkinsonism is also observed with other virus types. Experimental infection by H5N1 in mice show that the virus travels from the peripheral to central nervous system causing microglial activation, α-synuclein aggregation, and death of nigral dopaminergic neurons [155]. Traumatic brain injuries are also known to cause profound brain inflammation [156, 157], and in addition to increase in the levels of α-synuclein in the brain [158–160], it also increases the risk of PD [160, 161], further supporting the connection between inflammation and PD. Despite the fact that many studies support the role of inflammation in the process of neurodegeneration in PD and in other neurodegenerative diseases as well [144, 145, 162], the attention has been lately channeled into searching the role of disease-mutated proteins in PD, which can give clues about the pathophysiology of the disease but can be misleading if analyzed independently.

Post-mortem analysis of PD brains shows important microglial activation and glial invasion in areas where dopaminergic neurons degenerate [163, 164]. Even though inflammation and glial activation may be regarded as a secondary process [165], several studies in the literature support a more direct participation of inflammation in PD. First, proinflammatory molecules, including TNFα, IL-1β, and IL-6, and several toll-like receptors (TLRs) are elevated in PD brains [166–169]. Second, injection of PD-related toxins in experimental models, including MPTP and 6-hydroxydopamine, causes pronounced inflammation that leads to degeneration of dopaminergic neurons [169–171]. Injection of the TLR4 agonist, lipopolysaccharide (LPS), into the nigrostriatal pathway of rodents expressing normal or elevated levels of α-synuclein is associated with degeneration of dopaminergic neurons in the substantia nigra [172, 173], which is abated in mice that lack α-synuclein. Importantly, LPS injection also induces accumulation of intracellular insoluble aggregated α-synuclein [173]. LPS injection also increases the vulnerability of dopaminergic neurons to rotenone and 6-hydroxydopamine [152, 174] in a mechanism that involves the activation of several proinflammatory cytokines, in particular, IL-1β [152, 175]. In agreement, administration of IL-1 receptor antagonists significantly reduces TNF-α and IFN-γ levels, and attenuates the death of dopaminergic neurons caused by the LPS-induced sensitization at non-toxic doses [152]. In an additional inflammatory model, injection of the TLR3 agonist polyinosinic:polycytidylic acid [poly(I:C)] to the substantia nigra leads to sustained inflammation, increased susceptibility to low doses of 6-hydroxydopamine, and interfered with proteins involved with synaptic transmission and axonal transport [176]. In PD brain, TLR2 expression is elevated and correlates with accumulation of SDS-soluble α-synuclein [168] and TLR9 is also elevated in the brain of patients with PD compared to controls [169]. Substantia nigra dopamine degeneration induced by MPTP in mice is blocked by TLR9 deficiency [177].

In the cell biological lipid framework, knockout of GBA in zebrafish (which are devoid of α-synuclein) causes early microglial activation, reduction in motor activity, decrease of dopaminergic neurons by 30%, and the presence of intraneuronal ubiquitin-positive inclusions [178], supporting a role for inflammation in lipidopathy. Systemic administration of the GCase inhibitor CBE in mice also causes pronounced inflammation and complement activation parallel to α-synuclein accumulation and toxicity [58, 59]. We recently showed that levels of GPNMB, which is associated with astrocytes, microglia, and macrophages, and modulation of neuroinflammatory responses [179–181], are increased in the substantia nigra of patients with PD [151]. Remarkably, in experimental models, levels of GPNMB in the brain are elevated following lipid elevations caused by CBE, but are not altered by pure α-synucleinopathy, suggesting a potential for a dominant role of lipid-induced degeneration in PD [151]. Subsequent to our finding of GPNMB elevations in PD, it has also been discovered that GPNMB is elevated in the brain and CSF of patients with sporadic AD, and that GPNMB levels increase with disease progression in distinct AD transgenic mouse models and primarily colocalize with a distinct population of microglial cells located around amyloid plaques [182]. GPNMB elevations have also been shown in the brains, CSF, and plasma of the lysosomal storage disorders’ Gaucher disease and Niemann-Pick type C [183–185], further supporting an association of GPNMB in glycolipid dysregulation.

### Converging inflammatory mechanisms in neurodegenerative diseases such as PD

In addition to the evident brain degeneration that most people think of as PD, in practically every patient there is significant involvement of the peripheral nervous system [56] and also evidence of inflammation in the gastrointestinal system, and potentially also in other organ systems such as the skin [186]. Clearly, PD genetics and aging is a cell biological problem that affects the whole body and can signal dysfunction and cause inflammatory reactions in regions that do not cause severe symptoms caused by neurodegeneration. In this context, there are a number of inflammatory molecules and cascades associated with the colitis like syndrome that is typically seen in many PD patients [187]. It is also possible to obtain evidence of lipidopathy in epidermal skin cells from PD patients with GBA mutations [188].

GWAS of sporadic cases of multiple neurological diseases have identified risk genes that are associated with the immune system [189]. Moreover, several genes linked with familial neurodegenerative diseases are reported to have roles within the immune system [146, 190–193], for example, LRRK2 and α-synuclein in PD. The familial PD-associated gene, LRRK2, is expressed in both neurons and immune cells in human brain, and peripheral myeloid cells express LRRK2 at high levels, and the expression of LRRK2 is upregulated by inflammatory signals [194–197]. Overexpression of α-synuclein in transgenic mice can promote inflammation. Mice overexpressing 5–15 times α-synuclein under a hamster prion promoter have a clear motor phenotype and gliosis but no formation of Lewy body-like inclusions or neuronal degeneration [198], and mice overexpressing α-synuclein under the Thy1 promoter show microglial activation [199]. Interestingly, Thy1-α-synuclein overexpressing mice also express human α-synuclein in the peripheral nervous system as well the brain, and these mice exhibit autonomic dysfunction [200]. Also, while intracerebral injection of fibrillated α-synuclein promotes the accumulation of aggregated α-synuclein in the brain of α-synuclein transgenic and non-transgenic mice [201, 202], a key event is the protracted robust inflammation in the brain of injected mice, even in non-transgenic mice, where the accumulation of aggregated α-synuclein is less clear [203]. This evidence taken together supports that inflammation may in some cases precede the substantial accumulation of pathological α-synuclein inclusions, and that the α-synuclein per se may further induce the inflammatory process. Whereas α-synuclein (and beta and gamma) are normally found primarily on synaptic vesicles, with pathological gain of function, including lipidation, the localization and distribution of α-synuclein can be altered potentially eliciting immune reactions. Supporting a role for α-synuclein in immune responses is the finding that silencing of endogenous α-synuclein in the substantia nigra also induces a neuroinflammatory response and degeneration of dopaminergic neurons [204]. In aging, a disruption of glial function with senescence is sufficient to create an inflammatory condition in hippocampal and other brain regions that may relate to significant functional memory deficits [149]. In summary, many pathological signals, including metabolic, mitochondrial dysfunction, and lipid abnormalities, are likely to contribute to, converge on, and perpetuate the inflammatory process in PD. These findings indicate that inflammatory processes may represent both an early and late event in the pathophysiology of PD. Even though the use of nonsteroidal anti-inflammatory drugs decrease the incidence of PD by 15% [205], the identification of markers to diagnose the disease at initial stages may help establish immune modulatory strategies for the disease.

## Concluding remarks

The knowledge and analysis presented in this article provide new opportunities for identifying, measuring, and treating distinct features of age-dependent neurological diseases such as PD and several types of dementia disorders, at both cellular and systemic levels. For PD, the classic pathological sign used to diagnose at post-mortem have changed very little since the original observations of Lewy bodies in the substantia nigra and loss of dopamine neurons. The PD motor syndrome is fully explained by the loss of function and synapses linked to the midbrain dopaminergic system. Nevertheless, beyond the identification of α-synuclein as part of the Lewy body and the genetic risk that rare forms of mutations or multiplication of α-synuclein creates in families, few additional cellular pathological changes have gained diagnostic prominence. A surprising observation from familial genetics has shown that mutations that impair function in GCase in one allele (haploinsufficiency) create PD rather than Gaucher disease which is associated with loss of function in both *GBA* alleles and an early onset and fatal lipid storage disease throughout many organs in the body. An important insight that explains much of PD pathology is that aging by itself leads to a phenocopy of the GBA deficiency in patients and in mouse models [18, 53]. Genetic data has also put a spotlight on additional lysosomal enzymatic function, where > 50% of PD patients carry a mutation in a lysosomal storage disorder gene [16]. This is consistent with biochemical perturbations of various glycolipid pathways leading to PD pathological changes [58, 108–114], and electron microscopic evidence shows that the Lewy body is in fact composed of cellular organelles, membranes, and lipids, together with α-synuclein [17]. Lipids and α-synuclein changes occur with age [18, 19, 53] in ways that simulate many of the molecular changes associated with genetic risk factors. Given that for PD research cellular and animal models reveal that lipid and α-synuclein can interact to modify synaptic and vesicular transport, this points to several common pathways and potential pathological gain of α-synuclein function that trigger and sustains the development of PD.

On the new horizon are observations and experiments that reveal how glial and neuron physiological interactions are critical for the disease progression and final pathology. Microglia can both initiate and drive the disease under certain conditions [144, 152, 176]. Moreover, dysfunctional astrocytes can play critical roles in the initiation and progression of the disease. In a global context outside the brain, it is now clear that neurodegenerative disease pathology is present in many peripheral and central nervous system circuitries at the same time, with significant symptoms to patients from each system [56]. Relevant to such systemic manifestations appears to be many immune regulatory changes that are apparent in tissues and cellular systems affected by the disease before and after symptoms. Similar to PD, it is now evident that maladaptive immune responses can drive AD pathobiology. Finally, it is important to obtain diagnostic biomarkers that mirror these mechanisms to treat the actual primary *causes* of cellular dysfunction, rather than down-stream reactions or end-stage pathology of amyloidogenic proteins.

## Abbreviations

AD: Alzheimer’s disease
ER: Endoplasmic reticulum
GCase: Glucocerebrosidase
GPNMB: Glycoprotein NMB
IL-1β: Interleukin-1 beta
IL-6: Interleukin-1 6
LBD: Lewy body dementia
LPS: Lipopolysaccharide
PD: Parkinson’s disease
TLR: Toll-like receptor
TNFα: Tumor necrosis factor alpha
TREM2: Triggering receptor expressed on myeloid cells 2
